# The Construction of a Risk Prediction Model Based on Neural Network for Pre-operative Acute Ischemic Stroke in Acute Type A Aortic Dissection Patients

**DOI:** 10.3389/fneur.2021.792678

**Published:** 2021-12-23

**Authors:** Hongliang Zhao, Ziliang Xu, Yuanqiang Zhu, Ruijia Xue, Jing Wang, Jialiang Ren, Wenjia Wang, Weixun Duan, Minwen Zheng

**Affiliations:** ^1^Department of Radiology, Xijing Hospital, Fourth Military Medical University, Xi'an, China; ^2^GE Healthcare China, Beijing, China; ^3^Department of Cardiovascular Surgery, Xijing Hospital, Fourth Military Medical University, Xi'an, China

**Keywords:** aortic dissection, acute ischemic stroke, angiography, risk assessment, deep neural network, acute type A aortic dissection

## Abstract

**Objective:** To establish a pre-operative acute ischemic stroke risk (AIS) prediction model using the deep neural network in patients with acute type A aortic dissection (ATAAD).

**Methods:** Between January 2015 and February 2019, 300 ATAAD patients diagnosed by aorta CTA were analyzed retrospectively. Patients were divided into two groups according to the presence or absence of pre-operative AIS. Pre-operative AIS risk prediction models based on different machine learning algorithm was established with clinical, transthoracic echocardiography (TTE) and CTA imaging characteristics as input. The performance of the difference models was evaluated using the receiver operating characteristic (ROC), precision-recall curve (PRC) and decision curve analysis (DCA).

**Results:** Pre-operative AIS was detected in 86 of 300 patients with ATAAD (28.7%). The cohort was split into a training (211, 70% patients) and validation cohort (89, 30% patients) according to stratified sampling strategy. The constructed deep neural network model had the best performance on the discrimination of AIS group compare with other machine learning model, with an accuracy of 0.934 (95% CI: 0.891–0.963), 0.921 (95% CI: 0.845–0.968), sensitivity of 0.934, 0.960, specificity of 0.933, 0.906, and AUC of 0.982 (95% CI: 0.967–0.997), 0.964 (95% CI: 0.932–0.997) in the training and validation cohort, respectively.

**Conclusion:** The established risk prediction model based on the deep neural network method may have the big potential to evaluate the risk of pre-operative AIS in patients with ATAAD.

## Introduction

Pre-operative acute ischemic stroke (AIS) is one of important factors that affects the outcomes of surgical treatment and long-term post-operative survival in patients with acute type A aortic dissection (ATAAD) (1). Although ATAAD is a serious aortic disease that requires immediate surgical repair once diagnosed (1, 2), The treatment of ATAAD with pre-operative AIS is more challenging. Many studies had proven that neurological damage caused by pre-operative AIS or cerebral malperfusion indicates a poor prognosis following ATAAD (3, 4). In order to avoid the deterioration of AIS in patients with ATAAD, early preventive intervention during surgery is needed.

Computed tomography angiography (CTA) and transthoracic echocardiography (TTE) have shown high sensitivity in the evaluation of high-risk plaque and vascular anatomy (5), and thus, are commonly used methods for the diagnosis of aortic dissection (6). Some studies have suggested about 5–10% of patients with ATAAD had an AIS before surgery, which was caused by the extension of the dissection into the common carotid arteries, thromboembolism or cerebral hypoperfusion (2, 4, 7).

Neural network has become a popular algorithm for medical data analysis, especially for classification tasks, because it allows us to solve quite complex classification tasks (8, 9). Unfortunately, medical classification tasks usually have to be performed under more or less limited conditions, involving many cases in the data set, i.e., their size and uneven distribution between disease categories. One option is to reduce the majority class to the size of the minority class. This will make the training set too small and lead to unsuccessful machine learning. Neural network has many training optimization methods. For example, using an appropriate loss function can effectively solve the problem of sample imbalance (10).

Our previous study using univariate and multivariate analysis found that the true lumen diameter ratio of the ascending aorta (aAO), the common carotid artery (CCA) dissection, and the aortic valve insufficiency may be associated with pre-operative AIS (11). However, traditional univariate and multivariate analyses do not consider the information between characteristics, and thus, the corresponding logistic model in did not perform better in our previous study. Therefore, a risk prediction model used to accurately predict the pre-operative AIS in patients with ATAAD using deep neural network, which can deeply fuse the information among characteristics, will be constructed to help doctor make informed treatment decisions.

## Materials and Methods

### Study Population and Definitions

Between January 2015 and February 2019, a total of 300 ATAAD patients, diagnosed by aortic CTA and with no history of ischemic stroke or cerebrovascular disease, were retrospectively included in this study. ATAAD patients were divided into two groups according to the presence (AIS+ group) or absence (AIS- group) of pre-operative AIS. Clinical characteristics, TTE imaging information and CTA imaging information were collected. Detailed information was shown in Table 1 (clinical and TTE) and Table 2 (CTA).

**Table 1:** The clinical characteristic of patients with ATAAD.

**Characteristics**	**AIS + *n* = 86**	**AIS – *n* = 214**	***P***
**Demographics**		
Sex (male)	65 (75.6)	180 (84.1)	0.118
Age (year)	52.5 ± 9.8	48.1 ± 10.5	0.001^*^
**Medical history**		
Hypertension	61 (70.9)	134 (62.6)	0.218
Marfan's syndrome	1 (1.2)	2 (0.9)	0.638
Diabetes	1 (1.2)	3 (1.4)	0.676
Coronary heart disease	3 (3.5)	14 (6.5)	0.229
**Clinical symptoms**		
Chest pain	18 (20.9)	47 (22.0)	0.967
Back pain	12 (14.0)	30 (14.0)	0.999
Chest and back pain	21 (24.4)	84 (39.3)	0.021^*^
Abdominal pain	17 (19.8)	45 (21.0)	0.931
**Emergency examination**		
Systolic blood pressure	133 [99.2;146]	136 [116;154]	0.040^*^
Diastolic blood pressure	66.5 [55.0;77.8]	71.0 [60.0;84.8]	0.034^*^
Hypotension	12 (14.0)	11 (5.1)	0.019^*^
Malperfusion	17 (19.8)	27 (12.6)	0.050
Tamponade	10 (11.6)	10 (4.7)	0.029^*^
**Transthoracic echocardiography**		
AVI (moderate or severe)	22 (25.6)	46 (21.5)	0.268
LVEF	48.5 ± 5.8	49.2 ± 5.9	0.332
**Time interval**		
From symptoms onset to MR examination (h)	16.2 (8–50)	10 (6–23)	0.142

*ATAAD, acute type A aortic dissection; AIS, acute ischemic stroke; AVI, aortic valve insufficiency; LVEF, left ventricular ejection fraction*.

* *represents the statistical differences*.

**Table 2:** The CTA imaging characteristic information of patients with ATAAD.

**Characteristics**	**AIS + (*n* = 86)**	**AIS – (*n* = 214)**	***P***
The diameter of aAO	48.0 [44.0;52.0]	47.0 [43.0;50.8]	0.373
The true lumen diameter of aAO	12.0 [6.47;16.0]	17.0 [13.0;22.8]	<0.001^*^
The true lumen diameter ratio of aAO	0.24 [0.15;0.32]	0.36 [0.26;0.48]	<0.001^*^
The false lumen thrombus of aAO	23 (26.7)	68 (31.8)	0.473
Retrograde aAO dissection	11 (12.8)	31 (14.5)	0.843
Intimal flap plaque	30 (34.9)	39 (18.2)	0.003^*^
Entry tear in the aortic arch	41 (47.7)	123 (57.5)	0.157
CCA dissection	67 (77.9)	62 (29.0)	<0.001^*^
Innominate artery or CCA from false lumen	12 (14.0)	7 (3.3)	0.001^*^
Low density of unilateral ICA	24 (27.9)	13 (6.1)	<0.001^*^
VA dissection	1 (1.2)	3 (1.4)	0.676
VA from false lumen	5 (5.8)	5 (2.3)	0.125
VA from aortic arch	1 (1.2)	5 (2.3)	0.448
Low density of unilateral VA	8 (9.3)	6 (2.8)	0.021^*^
SA dissection	32 (37.2)	37 (17.3)	<0.001^*^
SA from false lumen	3 (3.5)	2 (0.9)	0.144
VSACV	0 (0.00)	2 (0.9)	0.508

*ATAAD, acute type A aortic dissection; AIS, acute ischemic stroke; aAO, ascending aorta; CCA, common carotid artery; ICA, internal carotid artery; VA, vertebral artery; SA, subclacian artery; VSACV, vagal subclavian artery congenital variation*.

* *represents the statistical differences*.

ATAAD was defined as any non-traumatic dissection of the aorta proximal to the left subclavian artery presenting within 14 days of symptom onset (1). All included ATAAD patients in this study are involved with ascending aorta as well as a primary entry tear either in the ascending aorta or the aortic arch (prior to the left subclavian artery). AIS was defined as a cerebrovascular accident representing a loss of neurological function (loss or slurring of speech, altered state of consciousness) caused by an ischemic event and further confirmed by diffusion MRI. Because the MRI examination room was adjacent to CT examination room in our emergency department and only cranial DWI sequence was performed, the total examination time was <5 min.

This study complied with the Helsinki Declaration (2000) and was approved by the institutional review board of Xijing Hospital affiliated with the Fourth Military Medical University (20120216-4). Informed consent was obtained from each patient or their legal representative.

### Aortic CTA

CTA examinations were performed using a second-generation dual source CT machine (Somatom Definition Flash; Siemens Healthcare, Forchheim, Germany) with a high-pitch spiral scan mode. Patients underwent combined CTA imaging of the neck and aorta in the cranio-caudal direction. For all scans, patients were in a supine position with both arms raised. Each patient received an injection of 70 ml of iopromide (Ultravist 370, 370 mgI/mL; Bayer Schering Pharma, Berlin, Germany) at a flow rate of 5 mL/s, followed by 40 mL of saline solution at a flow rate of. Bolus tracking was performed on the suprarenal descending aorta with an attenuation threshold of 100 HU. The scanning parameters were as follows: tube voltage of 100 kV, reference tube current of 300 mAs per rotation, pitch of 3.0 and slice collimation of 2 × 128 × 0.6 mm by means of a z-flying focal spot. The CTA images were transferred to an external workstation (syngo MMWP VE 36 A; Siemens Healthcare, Forchheim, Germany) for further postprocessing. Finally, CTA characteristics were identified from each patient CTA imaging by two experts with at least 10 years' experience on radiology imaging.

### Deep Neural Network

**Figure 1:**
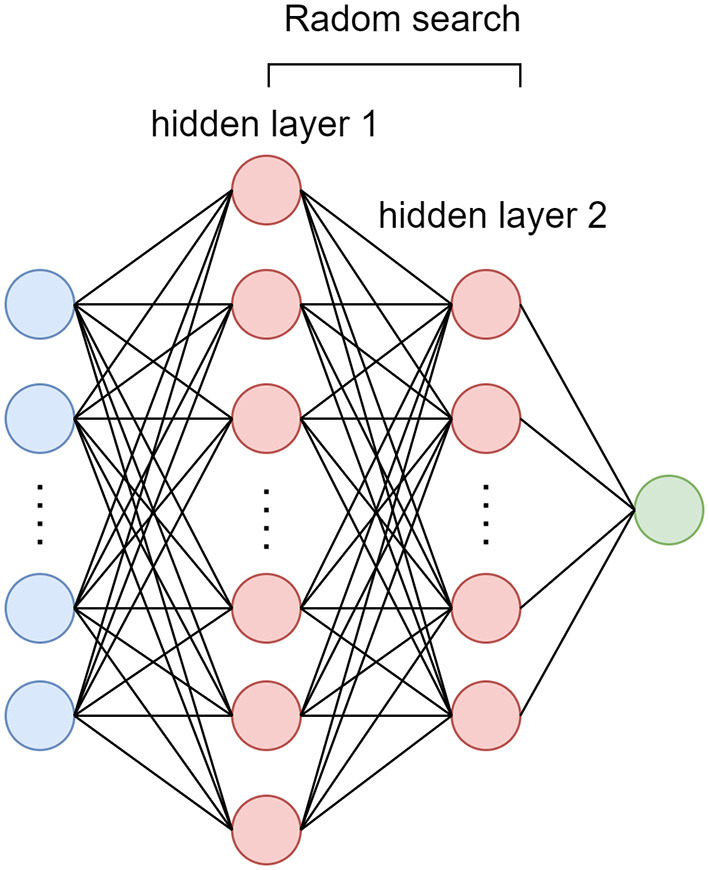
The model architecture of the deep neural network.

In this study, dataset is stratified split into training (70%) and validation (30%) cohort. A four layers deep neural network was performed with all the clinical, TTE and CTA imaging characteristics as input. The random search method (12) was used to select the best number of nodes for each hidden layer and the number of nodes for output layer was set to 1 represent AIS+ probability (Figure 1). In order to overcome the influence of data imbalance focal loss (10) were used as loss function as shown in equation (1). The Adam optimizer was used and model was trained with 300 epochs.


(1) $$loss=\left\{ \begin{array}{l} -{\left(1-\hat{p}\right)}^{\gamma }\log\left(\hat{p}\right)\text{ if }y=1\\ -{\hat{p}}^{\gamma }\log\left(1-\hat{p}\right)\text{ if }y=0 \end{array}\right.$$

Whereγ is a adjust factor andγ>0

### Machine Learning Model

In addition, the univariate and multivariate stepwise logistic regression analysis (Uni + Multi analysis) (11), the 10-fold CV based Least absolute shrinkage and selection operator (CV LASSO) risk factor selection method (13), support vector machine (SVM) model (14), random forest (RF) model (15), and neural network (NN) were also used for comparison. According to the number of input characteristics, the number of NN's nodes for each hidden layer were set as 16, 8, and 4, respectively. The number of nodes for output layer was set to 2, the same as the number of patient's group. The deep belief network (DBF) (16) training method was used to initialize the weights of the NN. Briefly, the first four layers of NN consisted of three restricted Boltzmann machines (RBMs). When pervious RBM finished the training, the trained weights would be used as the initialized weights for the corresponding NN layer, and the output of this trained RBM would be used as the input for the current RBM training.

For Uni + Multi analysis model, characteristics with significant difference between AIS+ and AIS- group in univariate model were included in the construction of multivariate stepwise logistic regression model. For CV LASSO model, all variables were selected through CV LASSO algorithm. For SVM, RF, NN and DNN model, all variables were included in the construction of the model. The first difference between NN and DNN was that the number of nodes for each hidden layer in NN was manually set according to number of input characteristics, while, for DNN, these number was optimized through random search method, which could make the performance of model to the best. The second difference was the weight initialization (DBF training method for NN and random initialization for DNN).

### Statistical Analysis

The training and validate of the deep neural network and DBN were implemented using TensorFlow 2.0 with GPU support on a Python 3.8 platform (https://www.python.org/). The other conventional machine learning model was implemented with R software (Version 4.1.0, https://www.rproject.com/).

All statistical analysis was performed using R software. Summary statistics are presented as counts with percentages for categorical values, as mean ± standard deviation for normally distributed continuous variables, or as medians with quartiles for non-normally distributed continuous variables. Data distribution was checked using the Shapiro-Wilk test. Continuous variables with normal data distributions were compared using two-sample *t* tests. For data with skewed distributions, non-parametric Mann-Whitney tests were used. Categorical variables were compared using chi-square statistics, and the Fisher exact test was used if observed frequencies were <5. Receiver operating characteristic (ROC) curve as well as precision recall curve (PRC) analysis and the area under the curve (AUC) was calculated. The best cutoff point was obtained by the Youden-index of ROC curve, then accuracy, sensitivity, specificity, positive predictive rate, and negative predictive rate were calculated. To estimate the clinical usefulness of the different models, decision curve analysis (DCA) was conducted by calculating the net benefits at different threshold probabilities (17). For all statistical analyses, *p* < 0.05 was considered statistically significant.

## Results

### Patient Clinical and TTE Imaging Characteristics

Pre-operative AIS was detected in 86 of 300 patients with ATAAD (28.7%). Among clinical and TTE imaging characteristics, the age (52.5 ± 9.8 years vs. 48.1 ± 10.5 years, *p* = 0.001), the incidence of hypotension (14.0 vs. 5.1%, *p* = 0.019) and tamponade (11.6 vs. 4.7%, *p* = 0.029) were significantly higher in the AIS+ group compared to the AIS- group, the systolic blood pressure (129.3 ± 34.0 vs. 137.5 ± 31.4 ml/Hg, *p* = 0.040) and the incidence of the chest and back pain (24.4 vs. 39.3%, *p* = 0.021) and were significantly lower in the AIS+ group compared to the AIS- group. Other clinical characteristics did not differ significantly between the AIS+ group and AIS- group. Detailed clinical and TTE imaging characteristics are shown in Table 1.

### Patient CTA Imaging Characteristics

Among CTA imaging characteristics, the true lumen diameter of the aAO (11.9 ± 5.9 vs. 17.8 ± 7.3 mm, *p* < 0.001) and the true lumen diameter ratio of the aAO (0.25 ± 0.11 vs. 0.37 ± 0.15, *p* < 0.001) were lower in the AIS+ group than in the AIS- group. The intimal flap plaque (34.9 vs. 39.0%, *p* = 0.003), the CCA dissection (77.9 vs. 29.0%, *p* < 0.001), the innominate artery or CCA from false lumen (14.0 vs. 3.3%, *p* = 0.001), the low density of the unilateral ICA (27.9 vs. 6.1%, *p* < 0.001), the low density of vertebral artery (9.3 vs. 2.8%, *p* < 0.021), and the subclavian artery dissection (37.2 vs. 17.3%, *p* < 0.001) were higher in the AIS+ group than in the AIS- group. Other CTA imaging characteristics did not differ significantly between the AIS+ group and AIS- group. Detailed CTA imaging characteristics are shown in Table 2.

### Model Performance

**Figure 2:**
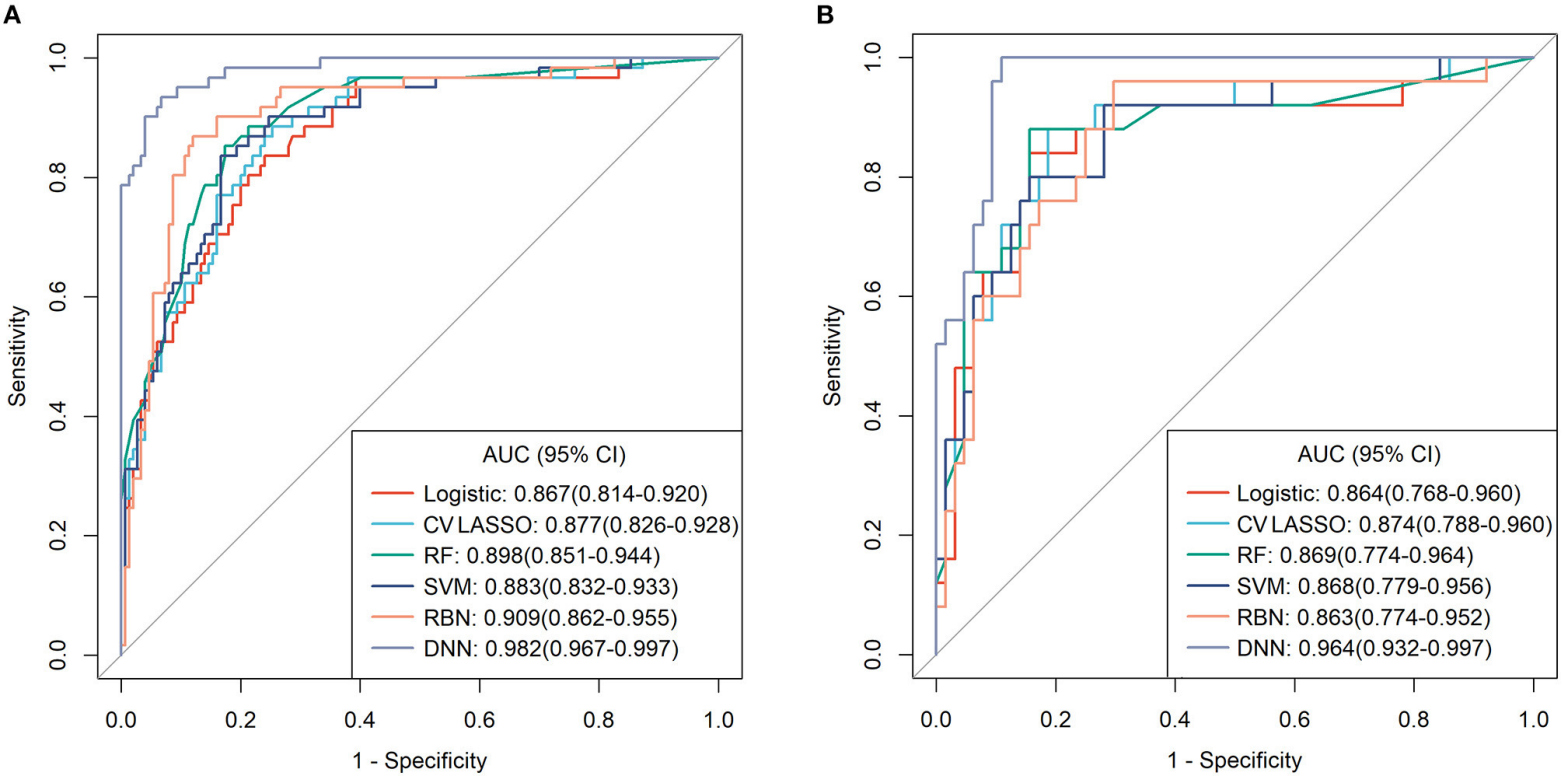
The receiver operating characteristic (ROC) curves for each model in **(A)** training cohort and **(B)** validation cohort.

**Figure 3:**
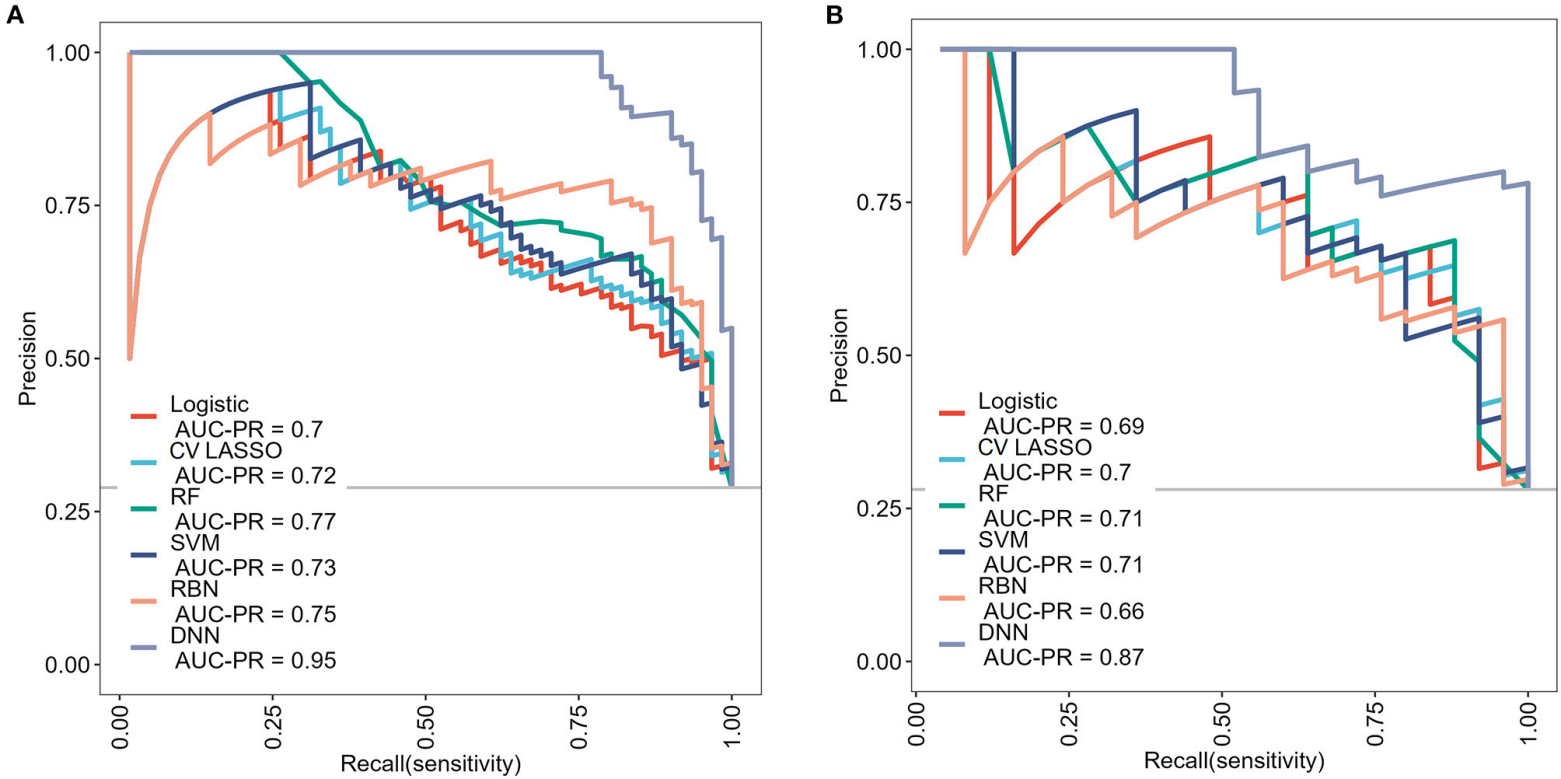
The precision-recall curves (PRC) for each model in **(A)** training cohort and **(B)** validation cohort.

**Figure 4:**
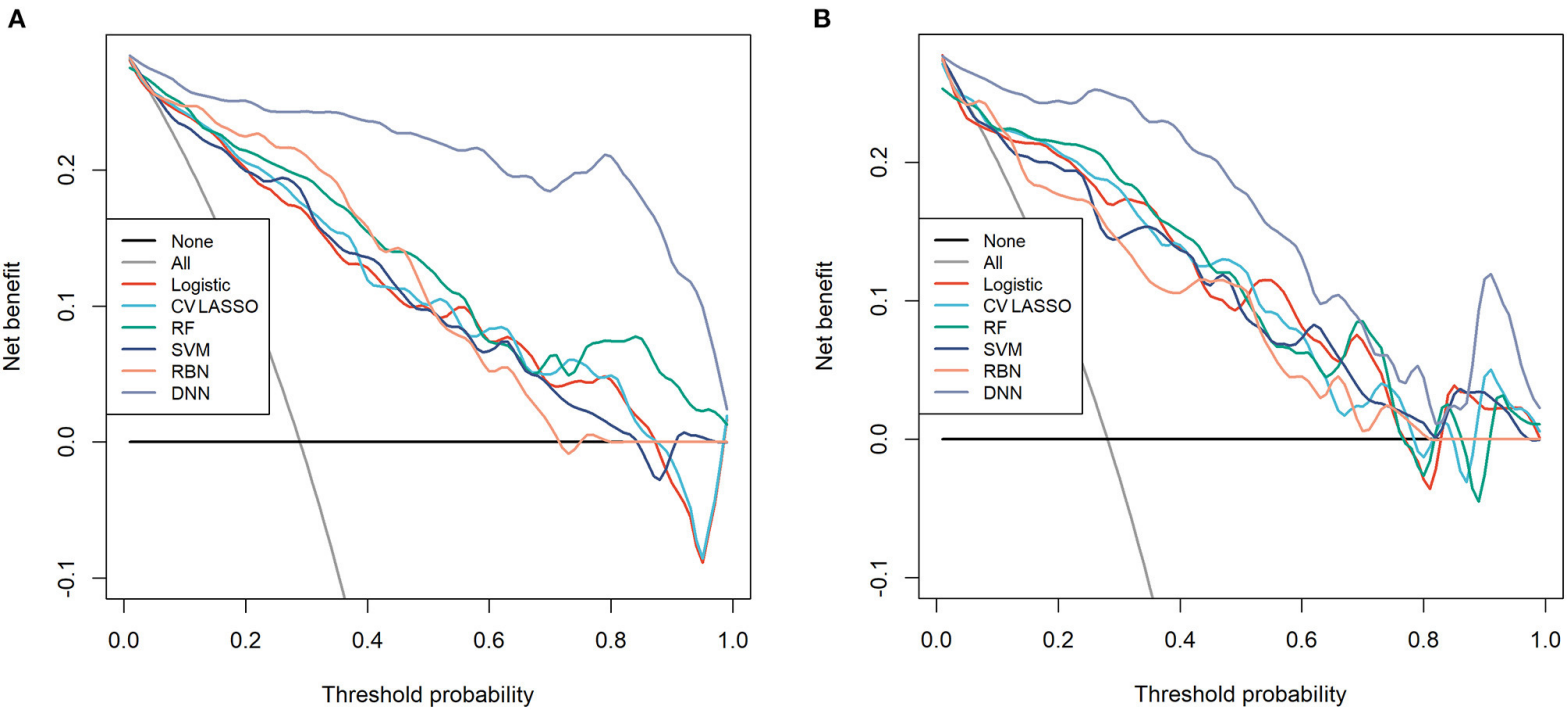
The decision curve analysis (DCA) for each model in **(A)** training cohort and **(B)** validation cohort.

Among those methods, the model constructed by the deep neural network had the best performance than the other methods, with an accuracy of 0.934 (95% CI: 0.891–0.963), 0.921 (95% CI: 0.845–0.968), sensitivity of 0.934, 0.960, specificity of 0.933, 0.906, and AUC of 0.983 (95% CI: 0.967–0.997), 0.964 (95% CI: 0.932–0.997) in the training and validation cohort, respectively (Table 3). The ROC curves were shown in Figure 2 and the best cutoff point of deep neural network was 0.402. The PRC as illustrated in Figure 3, which demonstrated that deep neural network has the best AUC with highest precision (positive predict rate) and recall (sensitivity). The DCA curve for the different models were presented in Figure 4. The DCA showed that if the threshold probability between 0.05 and 0.694, using the deep neural network to predict AIS status adds more net benefit than either the other models, “treat all patients” or the “treat none” strategies.

**Table 3:** The performance of different prediction models.

**Method**	**Training**						**Validation**					
	**AUC**	**ACC**	**SEN**	**SPE**	**PPV**	**NPV**	**AUC**	**ACC**	**SEN**	**SPE**	**PPV**	**NPV**
Uni + Multi analysis	0.867 (0.814–0.920)	0.782 (0.720–0.836)	0.836	0.760	0.586	0.919	0.864 (0.768–0.960)	0.798 (0.699–0.876)	0.840	0.781	0.600	0.926
CV LASSO	0.877 (0.826–0.928)	0.787 (0.725–0.840)	0.885	0.747	0.587	0.941	0.874 (0.788–0.960)	0.798 (0.699–0.876)	0.880	0.766	0.595	0.942
RF	0.898 (0.851–0.944)	0.834 (0.777–0.882)	0.852	0.827	0.667	0.932	0.869 (0.774–0.964)	0.843 (0.750–0.911)	0.840	0.844	0.677	0.931
SVM	0.883 (0.832–0.933)	0.834 (0.777–0.882)	0.836	0.833	0.671	0.926	0.868 (0.779–0.956)	0.753 (0.650–0.838)	0.800	0.734	0.541	0.904
DBN	0.909 (0.862–0.955)	0.877 (0.825–0.918)	0.869	0.880	0.746	0.943	0.863 (0.774–0.952)	0.809 (0.712–0.885)	0.720	0.844	0.64	0.885
DNN	0.982 (0.967–0.997)	0.934 (0.891–0.963)	0.934	0.933	0.851	0.972	0.964 (0.932–0.997)	0.921 (0.845–0.968)	0.960	0.906	0.800	0.983

*Uni + Multi analysis, univariate and multivariate analysis; CV LASSO, cross validation based least absolute shrinkage and selection operator; RF, random forest; SVM, support vector machine; DBN, deep belief network; DNN, deep neural network; AUC, the area under the curve; ACC, accuracy; SEN, sensitivity; SPE, specificity; PPV, positive predictive rate; NPV, negative predictive rate*.

## Discussion

In this study, four potential risk factors related to pre-operative AIS in patients with ATAAD were found using the neural network method. After CV LASSO regression, we found the age, the true lumen diameter ratio of the aAO, the CCA dissection, the low density of unilateral ICA exhibited the highest importance to the four risk factors, which might suggest that these four characteristics were more important to the assessment of pre-operative AIS. The corresponding results were discussed in detail below.

Dumfarth et al. pointed out that the pre-operative infractions in ATAAD patients were more likely to occur in the right cerebral hemisphere, which was consistent with our observations (18). They also suggested that pre-operative neurologic dysfunction was an independent risk factor of post-operative neurologic injury (16). Thus, objective assessment of pre-operative stroke is very important. Many studies had proven that pre-operative cerebral malperfusion and AIS are serious complications of ATAAD, which indicate poor prognosis (3, 7, 19, 20).

In the construction of classifications or a prediction model, the univariate and multivariate analysis only takes those characteristics that have significant difference between AIS+ and AIS− group into account and do not consider the information between characteristics. Although the traditional machine learning based feature selection method, such as LASSO regression, SVM and RF, can consider the information between characteristics, this kind of method remains those characteristics that have relatively big contribution to the model. The NN method can make the most use of all the input characteristics, deeply take all the information between them into account, select and merge them into several factors that important to the classifications or prediction model (Table 3), and thus, had better performance than univariate and multivariate analysis and traditional machine learning based feature selection methods. For DNN method, it not only had the advantages of NN, but also achieved the optimization for the number of nodes in each hidden layer and considered the data imbalance problem, and thus, had the best performance than other methods.

This study had some limitations. Firstly, the data used in this study was from a single hospital. Future studies are needed that consider multi-center data to validate the reliability of our constructed risk model. Secondly, this study was a retrospective study, so dataset may contain some bias. Future studies are needed to collect larger amounts of perspective data to the validate results of this study and provide a more objective and scientific theoretical basis for the prevention and treatment of pre-operative AIS in patients with ATAAD. Thirdly, this study only looked at pre-operative data. Future study will use both pre- and post-operative data to further improve the risk model.

In summary, this study provided a risk prediction model using deep neural network method to predict the occurrence of pre-operative AIS in patients with ATAAD. This risk model can provide information that is useful for identifying which ATAAD patients are at high risk of AIS before surgery, and therefore, help doctors decide whether patients need follow-up examinations or surgery, and decide the best timing for it.

## Data Availability Statement

The raw data supporting the conclusions of this article will be made available by the authors, without undue reservation.

## Ethics Statement

The studies involving human participants were reviewed and approved by Xijing Hospital affiliated with the Fourth Military Medical University (20120216-4). The patients/participants provided their written informed consent to participate in this study.

## Author Contributions

HZ, WD, and MZ contributed to the conception and design. RX and JW contributed to the acquisition of data. ZX, JR, and WW contributed to the and analysis of data. HZ, ZX, and MZ contributed to the interpretation of the results. HZ and ZX contributed to the manuscript writing. YZ, WD, and MZ contributed to the manuscript reviewing. All authors contributed to the article and approved the submitted version.

## Funding

This study has received funding by the National Natural Science Foundation of China under Grant No. 81870218, the Subject Boosting Project of Xijing Hospital under Grant No. XJZT18ML20, and the Key Research and Development Plan of Shaanxi Province under Grant No. 2020SF-151.

## Conflict of Interest

JR and WW were employed by the company GE Healthcare China. The remaining authors declare that the research was conducted in the absence of any commercial or financial relationships that could be construed as a potential conflict of interest.

## Publisher's Note

All claims expressed in this article are solely those of the authors and do not necessarily represent those of their affiliated organizations, or those of the publisher, the editors and the reviewers. Any product that may be evaluated in this article, or claim that may be made by its manufacturer, is not guaranteed or endorsed by the publisher.
